# A Quality Initiative to Improve Appropriate Use of Initial Outpatient Echocardiography Among Pediatric Cardiologists

**DOI:** 10.1097/pq9.0000000000000313

**Published:** 2020-07-23

**Authors:** Erik L. Frandsen, Soultana Kourtidou, Joel S. Tieder, Erin Alberda, Brian D. Soriano

**Affiliations:** From the * Division of Pediatric Cardiology, Seattle Children’s Hospital, Seattle, Wash.; † Division of Pediatric Cardiology, Weill Cornell Medicine, New York, N.Y.; ‡ Division of Hospital Medicine, Department of Pediatrics, University of Washington and Seattle Children’s Hospital, Seattle, Wash.; § Department of Patient Safety, Seattle Children’s Hospital, Seattle, Wash.

## Abstract

**Introduction:**

Appropriate use criteria (AUC) guide initial transthoracic echocardiogram (TTE) use in outpatient pediatrics. We sought to improve pediatric cardiologist TTE ordering appropriateness (mean AUC score) with a quality improvement initiative.

**Methods:**

The outcome of interest was the prospective AUC score for all initial outpatient TTEs ordered between November 2016 and August 2017, categorized per the AUC: “appropriate” (score 7–9), “may be appropriate” (4–6), “rarely appropriate” (1–3). Interventions included a didactic review of 2014 AUC and participant documentation of AUC criteria for each TTE. Participants met quarterly to evaluate outcome, process, and balancing measures, intervention effectiveness, and to identify and mitigate barriers.

**Results:**

Twenty-two pediatric cardiologists participated. TTE appropriateness level before (n = 216) and after (n = 557) intervention was high. There was no significant difference in mean baseline and post-intervention AUC score (7.42 ± 1.87 versus 7.16 ± 2.87, *P* = 0.1), nor in TTE sensitivity (27% versus 25%, *P* > 0.1) as a balancing measure. Among baseline studies, 81% were “appropriate,” and 6% “rarely appropriate.” Among post-intervention studies, 76% were “appropriate,” and 11% “rarely appropriate.” Barriers identified to implementing AUC include TTE indications not specified by current AUC, expectations of referring provider or parent to perform TTE, consistent provider application of AUC, and ability of AUC to capture comprehensive clinical judgment.

**Conclusions:**

Although the mean AUC appropriateness level was high, we were able to identify significant barriers to the implementation of AUC. Future efforts should focus on the reduction of “rarely appropriate” TTE ordering.

## INTRODUCTION

Transthoracic echocardiography is a convenient, safe, and accurate imaging tool for delineating cardiac anatomy and function. It is the most commonly used imaging modality in pediatric cardiology clinics. Increasing trends in transthoracic echocardiogram (TTE) ordering over the past decade, and the high cost and low yield for TTE in common outpatient indications have led to initiatives focused on responsible TTE use.^1–3^ One such initiative is appropriate use criteria (AUC), which has been available for adults since 2007.^4^ Spurred by data from implementation studies of AUC, quality improvement (QI) projects in adult medicine have found success in improving TTE ordering appropriateness.^5–8^

In 2014, a multidisciplinary pediatric group published AUC for ordering initial TTE in the pediatric outpatient setting.^9^ The goal of this publication is to provide clinicians with a tool to improve patient care and health outcomes cost-effectively. The AUC publication provides 113 indications for TTE studies, rating each as “appropriate,” “may be appropriate,” or “rarely appropriate” with a corresponding score. Several implementation studies have evaluated the use of AUC in the pediatric clinical setting.^10–13^

The global aims of this initiative were to reduce the total costs and resource utilization associated with less-appropriate diagnostic testing. Our institution recently published an implementation study of AUC, wherein “rarely appropriate” indications comprised 14% of all TTE studies.^10^ As a result of this finding, the project aim was to improve overall TTE ordering appropriateness (ie, mean AUC score) by 10% through AUC based educational interventions. We also sought to identify barriers to implementing AUC.

## METHODS

### Context

This QI initiative took place in the Heart Center at Seattle Children’s Hospital, an academic tertiary medical center located in Seattle, WA. The Heart Center is comprised of more than 40 cardiologists with practice locations spanning 3 states. We invited cardiologists practicing at the main campus in Seattle, WA, to participate in the QI initiative. The echocardiography lab at the main campus performs nearly 900 initial outpatient TTEs yearly.^10^

A pediatric cardiologist (B.D.S.) lead the project. Seattle Children’s Maintenance of Certification (MOC) Portfolio program provided QI consultation (J.S.T.), and participants were eligible for MOC part 4 credit. The Seattle Children’s American Board of Medical Specialties Multispecialty MOC Portfolio Program, accredited in 2012, maintains a portfolio of approved MOC projects.

### Planning the Intervention

**Fig. 1.**
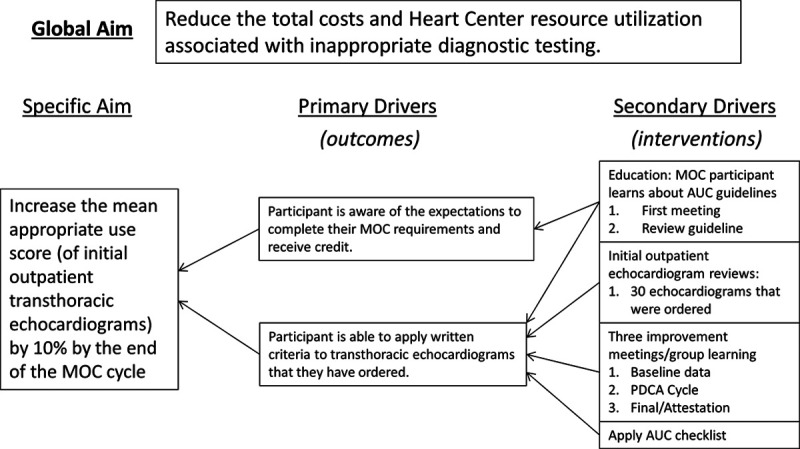
Key driver diagram.

This initiative aimed to increase the group’s mean AUC score by 10% above the baseline. Primary drivers identified were participant knowledge of the 2014 pediatric TTE AUC (Fig. 1). Secondary aims included applying AUC to TTE ordering and improving provider skills in QI through focused educational interventions, assessed by pre- and post-project surveys. The project leader met with MOC Portfolio consultants to develop educational interventions to address the primary drivers.

### Intervention

We obtained baseline data by reviewing the initial outpatient TTEs performed in our main campus echocardiography lab 4 months before the intervention. The 2014 TTE AUC encompasses initial outpatient TTE and assign scores to each indication. A score of 1–3 carries a rating of “rarely appropriate,” “may be appropriate” 4–6, and “appropriate” 7–9. In our study, as in prior AUC implementation studies, specific indications are not encompassed by the AUC and receive an “unclassifiable” rating. We excluded initial studies for patients with known heart disease.

**Fig. 2.**
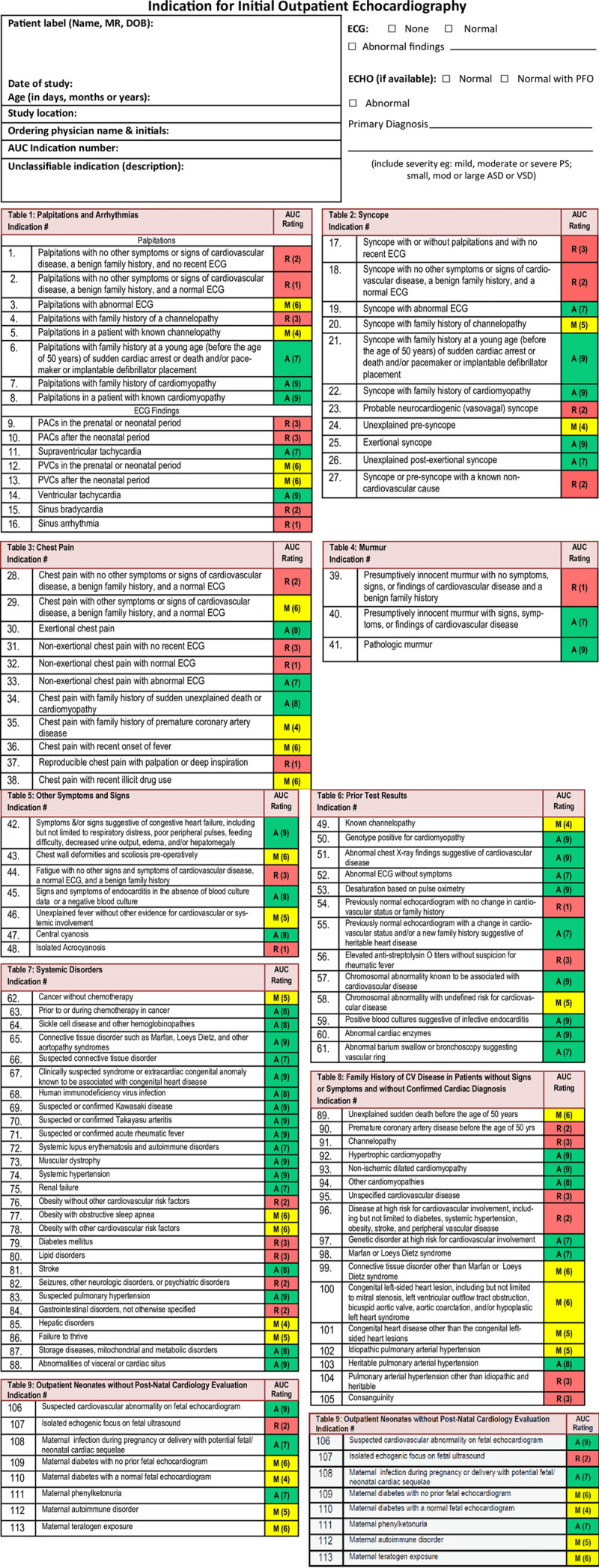
Handout given to participants at the first meeting detailing data elements required to be collected with each TTE study ordered as well as AUC reference tables (Courtesy of Dr. Ritu Sachdeva, Sibley Heart Center Cardiology).

We launched the QI initiative during an initial meeting in November 2016, where we reviewed the 2014 TTE AUC and presented baseline data. We provided participants with a one-page double-sided handout with a complete list of indications for initial outpatient TTEs and their respective rating as well as the list of data elements required for each study (Fig. 2). Participants were instructed to refer to the AUC handout and assign a rating for initial outpatient echocardiograms they ordered during the study period. Participants recorded data including electrocardiogram (ECG) findings, TTE findings, follow-up recommendations, and specifics of ordering indications in a RedCap database. If the TTE indication was not included in the AUC, participants assigned an “unclassifiable” rating and provided indication details. TTE studies rated as “unclassifiable” by the participant were reviewed by a secondary observer to ensure that the study indication was not included in the AUC. If a study was inappropriately rated as “unclassifiable,” the study was rated accurately and included in the statistical analysis and control chart. We held 2 quarterly meetings (March 2017 and June 2017), where we reviewed the outcome, process, and balancing measures using a statistical process control (SPC) chart. We evaluated interventional effectiveness and identified and mitigated barriers to the application of AUC to TTE ordering. Cardiologists were offered MOC part 4 credit as an incentive to participate in the QI initiative. To be eligible for MOC credit, attendance at the initial meeting and subsequent quarterly meetings were mandatory. In the instances where participants could not attend a quarterly meeting, the senior author (B.D.S.) met with individuals to review their progress. Additionally, participants were required to complete QI training, which was available by a variety of distance learning platforms (eg, Institute for Healthcare Improvement Open School). Each participant completed pre- and post-participation surveys (MOC-PEAKS) to assess the impact of the QI initiative and educational activities.^14^

### Outcome Assessment

The primary outcome was the average AUC score for initial outpatient TTE post-intervention compared to baseline. Secondary outcomes included the proportion of studies rated as “appropriate,” “may be appropriate,” and “rarely appropriate” post-intervention compared to baseline. As a balancing measure, we recorded abnormal findings from each TTE to evaluate for a reduction in sensitivity of TTE following our intervention. Abnormal findings excluded incidental isolated patent foramen ovale, left superior vena cava, and right aortic arch with mirror image branching.

### Data Analysis

Sample characteristics were summarized using appropriate descriptive statistics for quantitative and categorical variables. We calculated and compared the average baseline and post-intervention AUC scores using Student’s *t* test. To account for changes in ordering practices during the study period, we compared the mean AUC scores following the second quarterly meeting to baseline. We constructed a SPC chart with 1-month time intervals to visualize the effect of the intervention on AUC scores. The percentages of abnormal findings were compared at baseline and post-intervention using 2-proportion Z-test. We noted the percentage of unclassifiable indications at baseline and post-intervention. However, they were not included in descriptive or comparative statistics.

### Ethical Considerations

There were no ethical objections to this QI initiative. The Seattle Children’s Hospital Institutional Review Board approved this study and waived participant consent.

## RESULTS

Twenty-two cardiologists participated in the QI initiative, representing 73% of cardiologists who practice on the main campus. The baseline group consisted of 216 studies, with an average AUC score of 7.42 ± 1.87. Ninety-four percent of studies were classifiable by the AUC. Eighty-one percent (n = 175) of studies had an “appropriate” rating, 13% (n = 29) “may be appropriate,” and 6% (n = 12) “rarely appropriate.” The post-intervention group consisted of 557 studies. The average AUC score was 7.16 ± 2.87. Ninety-six percent of studies were classifiable. Seventy-six percent (n = 425) of studies had an “appropriate” rating, 13% (n = 71) “may be appropriate,” and 11% (n = 61) “rarely appropriate.”

**Fig. 3.**
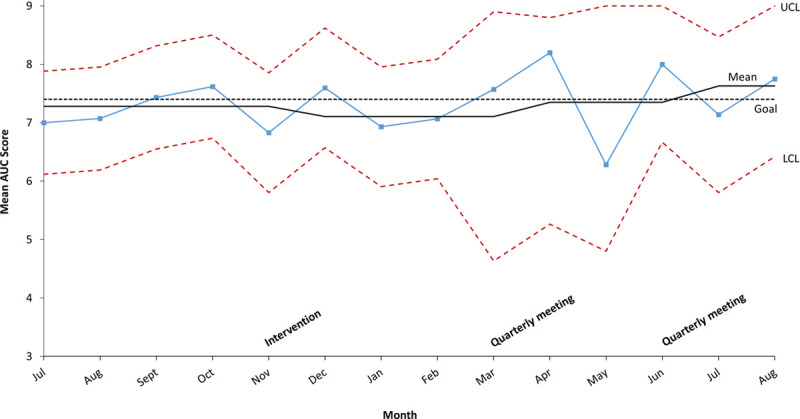
SPC chart of mean AUC score for initial outpatient transthoracic echocardiogram studies ordered before and after the initial intervention and after each subsequent quarterly meeting. LCL, lower control limit; UCL, upper control limit.

The difference between baseline and post-intervention mean AUC score did not reach statistical significance (*P* = 0.1). The mean AUC score for studies ordered after the second quarterly meeting was 7.4 ± 2.4, which was also not significantly different from baseline (*P* = 0.4). The SPC chart displays mean AUC scores by month throughout the QI initiative (Fig. 3).

Balancing measures included the proportion of abnormal findings from all studies. Table 1 shows the percentage of abnormal findings by AUC classification in the post-intervention group compared to baseline, of which there was no significant difference (27% versus 25%, *P* > 0.05). We found abnormal findings in 30%, 18%, and 15% of “appropriate,” “may be appropriate,” and “rarely appropriate” rated studies, respectively. Table 2 details the abnormal results from studies with “rarely appropriate” ratings, including ECG findings and follow-up recommendations. Abnormal findings included small muscular ventricular septal defect (n = 2), Secundum atrial septal defect (n = 2), semilunar valve abnormality without significant dysfunction (n = 4), and mild aortic arch hypoplasia (n = 1). The majority (78%) had a normal ECG. In 89%, follow-up was recommended. Presumptively innocent murmurs represented the most frequent (56%) “rarely appropriate” indication for TTE. We compared the proportion of abnormal studies in the post-intervention group across all AUC ratings. We found that “appropriate” studies had higher diagnostic yield compared to “may be appropriate” studies (30% versus 18%, *P* < 0.05) or “rarely appropriate” studies (30% versus 15%, *P* < 0.01). When “appropriate” and “may be appropriate” studies were grouped and compared to “rarely appropriate” studies, diagnostic yield remained significant (28% versus 15%, *P* < 0.05). Diagnostic yield between “may be appropriate” and “rarely appropriate” studies was not significantly different (18% versus 15%, *P* > 0.1).

**Table 1.**
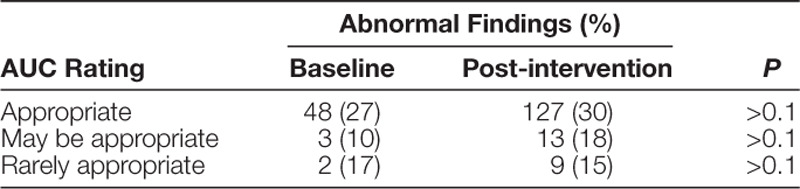
Abnormal Findings on Transthoracic Echocardiogram by AUC Indication at Baseline and Postintervention

**Table 2.**
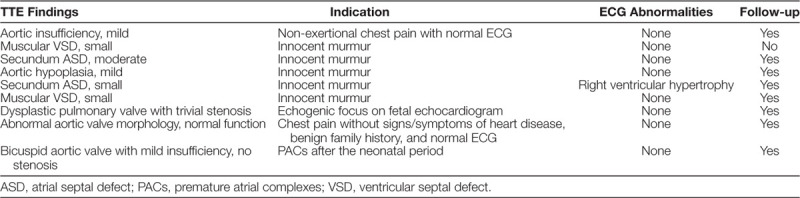
Abnormal Findings on Transthoracic Echocardiogram Studies With “Rarely Appropriate” Indication With Any Noted ECG Abnormalities and Recommendations for Follow-up

Indications for “rarely appropriate” studies were evaluated separately. Fifty-six percent of “rarely appropriate” TTE study indications were for a “presumptively innocent murmur with no symptoms, signs, or findings of cardiovascular disease, and a benign family history.” “Syncope with no other symptoms or signs of cardiovascular disease, a benign family history, and a normal ECG” and “chest pain with no other symptoms or signs of cardiovascular disease, a benign family history, and a normal ECG” each represented 7% of “rarely appropriate” studies. Other indications each represented less than 5% of all “rarely appropriate” studies.

Thirty-eight TTEs were initially given an “unclassifiable” rating, meaning the indication for the study did not fall under the 2014 TTE AUC. Each TTE indication was reviewed independently, and 14 were inappropriately categorized as “unclassifiable.” Eleven would have fallen in the “appropriate” category and 3 in the “may be appropriate” category. Two studies were assigned an “unclassifiable” rating for obstructive sleep apnea but did not specify the presence of obesity (if obese, this is an “appropriate” rating). Two studies were ordered to rule out a vascular ring; only one indicated that a barium swallow was abnormal (“appropriate” rating). Four orders were assigned an “unclassifiable” rating for presyncope with exertion (AUC give a “may be appropriate” rating to “unexplained presyncope”). The majority (n = 5) of unclassifiable indications were for abnormal heart sounds other than a murmur, including a click and split S1. Other indications included respiratory symptoms without heart failure findings, hemangioma, and history of chemotherapy.

## DISCUSSION

### Summary

The main finding from this QI initiative was that even at baseline, the mean AUC score was high (7.42), and 77% of study indications had an “appropriate” rating. We were not able to accomplish the primary aim of increasing the group mean AUC score by 10% from baseline, perhaps for this reason. In our cohort of pediatric cardiologists, an increase in the AUC score over time may not be a good metric to gauge improvement. This possibility is underscored by the fact that the baseline mean score was already within the “appropriate” (AUC score ≥ 7) range. An average score also does not highlight the percentage of studies ordered for “rarely appropriate” indications, which may have a low yield of abnormal findings, may lead to further unnecessary testing or patient/parental anxiety, and may not be reimbursed by insurance providers. Focusing QI efforts on reducing the number of studies that are “rarely appropriate” is a preferred approach. QI initiatives focusing on TTE AUC in adults have used a percent change in rating classes as markers of improvement.^6,15^

**Fig. 4.**
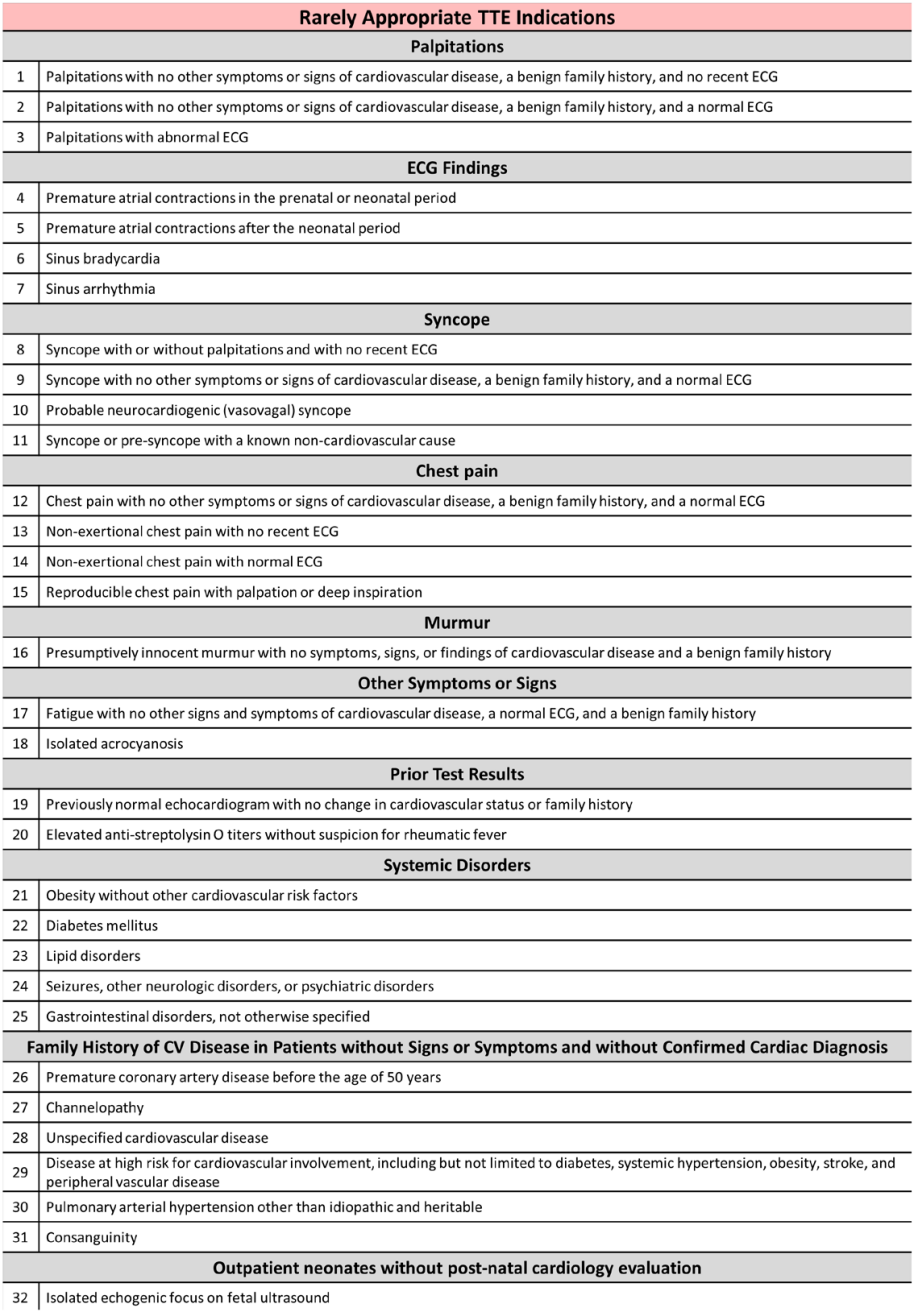
Proposed handout with a compiled list of “rarely appropriate” TTE indications according to the 2014 appropriate use criteria for initial transthoracic echocardiography in outpatient pediatric cardiology.^9^

Our study did not experience a reduction in “rarely appropriate” studies (6% baseline versus 11% post-intervention). Explanations for this finding may include this not being the primary aim of the initiative, and so participants did not pay as much attention to this while ordering TTE studies. Providing each individual with a handout listing “rarely appropriate” indications (Fig. 4) to guide non-testing may be more effective. The high percentage of “appropriate” rated indications (77%) may have also contributed to our failure to increase the average AUC score. The percentage of “appropriate” rated studies is similar to previously published TTE AUC implementation studies (71%–77%).^10–12^ Because this QI initiative was launched 1 year after the AUC publication, we cannot exclude whether the cohort’s exposure to the publication altered their ordering behavior before the baseline study. However, a recent study by Sachdeva et al^16^ would argue against a significant impact of the AUC publication on ordering behavior.

The application of AUC to TTE ordering was a secondary aim of this QI initiative. The finding that the AUC encompassed 36% of initially “unclassifiable” indications provides an opportunity for improvement in future PDSA (Plan-Do-Study-Act) cycles. Only TTE indications rated as “unclassifiable” were audited. In future QI, auditing and providing focused participant feedback may prove useful. At each quarterly meeting, the SPC chart was reviewed with the entire group, which may not have been as effective as providing each participant with their AUC score or AUC rating breakdown as a form of peer benchmarking.

### Participant Education and Feedback

Likert-scaled averages (data not shown) showed increased agreement with the statement, “the MOC experience is an important tool in improving the delivery of healthcare as well as the ability to use and interpret run charts to improve patient care, and the ability to apply QI methods to improve healthcare delivery for patients.” Despite the perceived obstacles of part 4 MOC,^17^ MOC is essential in demonstrating expertise in the field, keeping medical knowledge up to date, and demonstrating competency in providing medical care.^18^ Our study shows that multidisciplinary groups can create projects to capture the interest of subspecialty providers. Physician leaders can be coached by QI consultants to deliver quality projects that provide MOC part 4 credit.

This project was generally well-received among the participants. Reflections elicited were particularly telling of their experience. “I am more thoughtful about why I order an echo on a patient,” wrote one participant. Another noted that “I learned more factual information regarding practice patterns and the data supporting appropriate TTE indications.” Another went so far as to say, “the list of AUC for TTEs now lives on my home page and is something I refer to at each clinic to minimize unnecessary testing.” During the final meeting, participants discussed ways to integrate AUC into the daily workflow to reduce TTEs ordered for “rarely appropriate” indications. The group discussed the integration of AUC indications and corresponding ratings into the electronic medical system at the time of TTE ordering and discussed its feasibility. Point-of-care testing algorithms integrated into the electronic medical record system have been reported as modestly successful at improving the application of AUC.^19^ Participants also noted that referring provider and parental expectation for testing can be an influential factor in TTE ordering. This aspect was not explored in this QI initiative. A recent study showed that parental anxiety was reduced by performing a TTE for an innocent murmur, an indication considered “rarely appropriate” by the current AUC.^20^ Additionally, prior studies have shown a discrepancy in TTE ordering appropriateness between primary care providers and cardiologists.^10,21^ To what degree referring provider and parental/patient expectation of TTE does and should influence TTE ordering is an interesting question and deserving of future study, particularly in the setting of cost-conscious medicine and value-based reimbursement.

### Limitations of Current Appropriate Use Criteria

We reviewed TTE orders for indications not classified by the current AUC. From a review of the literature, a recurrent theme among unclassifiable indications is that for an abnormal heart sound other than a murmur, such as a valvular click or split first or second heart sound, which represented the majority (n = 5) of the unclassifiable indications for a TTE in our cohort. The diagnostic yield for TTE in “rarely appropriate” indications was surprising to us. We observed abnormal echocardiographic findings in 15% of studies assigned a “rarely appropriate” rating. None of these findings were critical, although the majority elicited cardiology follow up (Table 2). Other pediatric TTE AUC studies have observed diagnostic yield for “rarely appropriate” indications of 2%–9%.^10,11^ In our study, “appropriate” studies had higher diagnostic yield than “rarely appropriate” studies (30% versus 15%, *P* < 0.01), however, the diagnostic yield of “may be appropriate” and “rarely appropriate” studies were not significantly different (18% versus 15%, *P* > 0.1). The latter finding is quite interesting and questions the AUC distinction between indications that are considered “rarely appropriate” and those that “may be appropriate.” All abnormal results discovered during “rarely appropriate” TTE were mild, but follow up was recommended in 89%.

Several studies have reported on diagnostic yield by AUC appropriateness level. Diagnostic yield for “appropriate” rated TTE indications ranges from 20% to 23%, with overall yield ranging from 13% to 19%.^10,13^ In our study, diagnostic yield for “appropriate” TTE was 30%, which we suspect is because the most significant proportion of “appropriate” studies were for the indication “pathologic murmur.” An indication of pathologic murmur has been shown to have the highest yield of abnormal findings.^11,13^ Diagnostic yield of TTE also varies across age groups. Safa and colleagues examined the diagnostic yield of TTE across children of all ages. Age less than 1 year was a significant risk factor for having an abnormal finding (odds ratio 15, *P* < 0.001).^13^ We did not study diagnostic yield by age, but modifications to AUC in the future may consider including patient age when rating TTE appropriateness.

The most common indication for “may be appropriate” studies was a family history of congenital left-sided heart lesions, including mitral stenosis, left ventricular outflow tract obstruction, bicuspid aortic valve, aortic coarctation, and/or hypoplastic left heart syndrome, which represented 30% of these studies. A family history of sudden unexplained death before 50 years represented 17% of all “may be appropriate” study indications, and palpitations with abnormal ECG represented 13%. The most common indication for “rarely appropriate” studies was a presumptively innocent murmur, which represented 56% of all “rarely appropriate” studies. The distinction between innocent and potentially pathologic murmurs involves a degree of subjectivity on behalf of the practitioner, despite characteristics of pathologic murmurs published broadly.^22^ Additionally, the decision to perform an echocardiogram for a murmur most certainly considers other factors besides cardiac auscultation, including parental or referring provider expectations and clinical gestalt, which were not evaluated in this QI initiative but are deserving of future investigation. Differences in clinical opinion exist when addressing “innocent murmurs,” especially in infants, when murmurs may reflect a normal transition from fetal to postnatal physiology rather than pathology (such as in peripheral pulmonic stenosis). In this instance, rather than making the choice to perform or not perform a TTE, one may consider a follow-up in the future to relisten. It is difficult, if not impossible, to encompass every clinical decision in AUC. AUC seeks to be comprehensive to guide the practitioner’s use of cardiovascular tests, which the current AUC seems to do well.

## CONCLUSIONS

Although TTE appropriateness level before and after the intervention was high, we were able to identify significant barriers to implementation of AUC by pediatric cardiologists at an academic children’s hospital. Future interventions should focus on reducing “rarely appropriate” TTE studies, integrating AUC into the electronic medical system, and investigating factors outside of the history and physical that affect TTE ordering practices.

## DISCLOSURE

The authors have no financial interest to declare in relation to the content of this article.
